# Human Natural Killer Cell Maturation Defect Supports In Vivo CD56^bright^ to CD56^dim^ Lineage Development

**DOI:** 10.1371/journal.pone.0051677

**Published:** 2012-12-11

**Authors:** Carolina Inés Domaica, Mercedes Beatriz Fuertes, Ignacio Uriarte, María Victoria Girart, Jessica Sardañons, Dorina Ileana Comas, Daniela Di Giovanni, María Isabel Gaillard, Liliana Bezrodnik, Norberto Walter Zwirner

**Affiliations:** 1 Laboratorio de Fisiopatología de la Inmunidad Innata, Instituto de Biología y Medicina Experimental (IBYME), Consejo Nacional de Investigaciones Científicas y Técnicas (CONICET). Buenos Aires, Argentina; 2 Departamento de Química Biológica, Facultad de Ciencias Exactas y Naturales, Universidad de Buenos Aires, Buenos Aires, Argentina; 3 Unidad de Inmunología, Hospital de Niños “Ricardo Gutiérrez”. Buenos Aires, Argentina; 4 Departamento de Microbiología, Parasitología e Inmunología, Facultad de Medicina, Universidad de Buenos Aires, Buenos Aires, Argentina; University of Sydney, Australia

## Abstract

Two populations of human natural killer (NK) cells can be identified in peripheral blood. The majority are CD3^−^CD56^dim^ cells while the minority exhibits a CD3^−^CD56^bright^ phenotype. *In vitro* evidence indicates that CD56^bright^ cells are precursors of CD56^dim^ cells, but *in vivo* evidence is lacking. Here, we studied NK cells from a patient that suffered from a melanoma and opportunistic fungal infection during childhood. The patient exhibited a stable phenotype characterized by a reduction in the frequency of peripheral blood CD3^−^CD56^dim^ NK cells, accompanied by an overt increase in the frequency and absolute number of CD3^−^CD56^bright^ cells. These NK cells exhibited similar expression of perforin, CD57 and CD158, the major activating receptors CD16, NKp46, NKG2D, DNAM-1, and 2B4, as well as the inhibitory receptor CD94/NKG2A, on both CD56^bright^ and CD56^dim^ NK cells as healthy controls. Also, both NK cell subpopulations produced IFN-γ upon stimulation with cytokines, and CD3^−^CD56^dim^ NK cells degranulated in response to cytokines or K562 cells. However, upon stimulation with cytokines, a substantial fraction of CD56^dim^ cells failed to up-regulate CD57 and CD158, showed a reduction in the percentage of CD16^+^ cells, and CD56^bright^ cells did not down-regulate CD62L, suggesting that CD56^dim^ cells could not acquire a terminally differentiated phenotype and that CD56^bright^ cells exhibit a maturation defect that might result in a potential altered migration pattern. These observations, support the notion that NK cells of this patient display a maturation/activation defect that precludes the generation of mature NK cells at a normal rate accompanied by CD56^dim^ NK cells that cannot completely acquire a terminally differentiated phenotype. Thus, our results provide evidence that support the concept that *in vivo* CD56^bright^ NK cells differentiate into CD56^dim^ NK cells, and contribute to further understand human NK cell ontogeny.

## Introduction

Natural Killer (NK) cells exert cytotoxic functions and secrete IFN-γ and other pro-inflammatory cytokines against virus-infected and tumor cells. Also, NK cells are key regulators of the adaptive immune response through their cross talk with dendritic cells (DCs) that promotes DC maturation, and T helper (Th) 1- and cytotoxic T lymphocyte (CTL)-mediated immunity [1], [2]. NK cell activity is regulated by cytokines such as interleukin (IL)-2, IL-12, IL-15, IL-18 and type I interferons (IFNs) [3]. NK cell effector function is also triggered upon recognition of target cells through activating receptors such as NKG2D, DNAM-1, 2B4, the Natural Cytotoxicity Receptors (NCRs) NKp46, NKp44 and NKp30, and members of the Killer Immunoglobulin-like Receptor (KIR) family that carry a short cytoplasmic tail (KIR2DS and KIR3DS) [4], [5]. Conversely, normal cells are preserved from NK cell mediated functions because they promote engagement of inhibitory KIR receptors that carry a long cytoplasmic tail (KIR2DL and KIR3DL), CD94/NKG2A and members of the Immunoglobulin-Like Transcript (ILT) receptor family [4], [5]. Human NK cells, defined as CD3^−^CD56^+^ cells, can be subdivided in two main cell populations. About a 90% are cytotoxic CD56^dim^CD16^+^ cells, while the remaining 10% are CD56^bright^CD16^dim/−^ non-cytotoxic cells that are abundant in secondary lymphoid organs [6], [7]. This subpopulation expresses CD62L (L-selectin) and CCR7, which directs their homing to these niches but upon activation, CD62L down-regulation facilitates NK cell trafficking to inflamed tissues [8].

**Figure 1:**
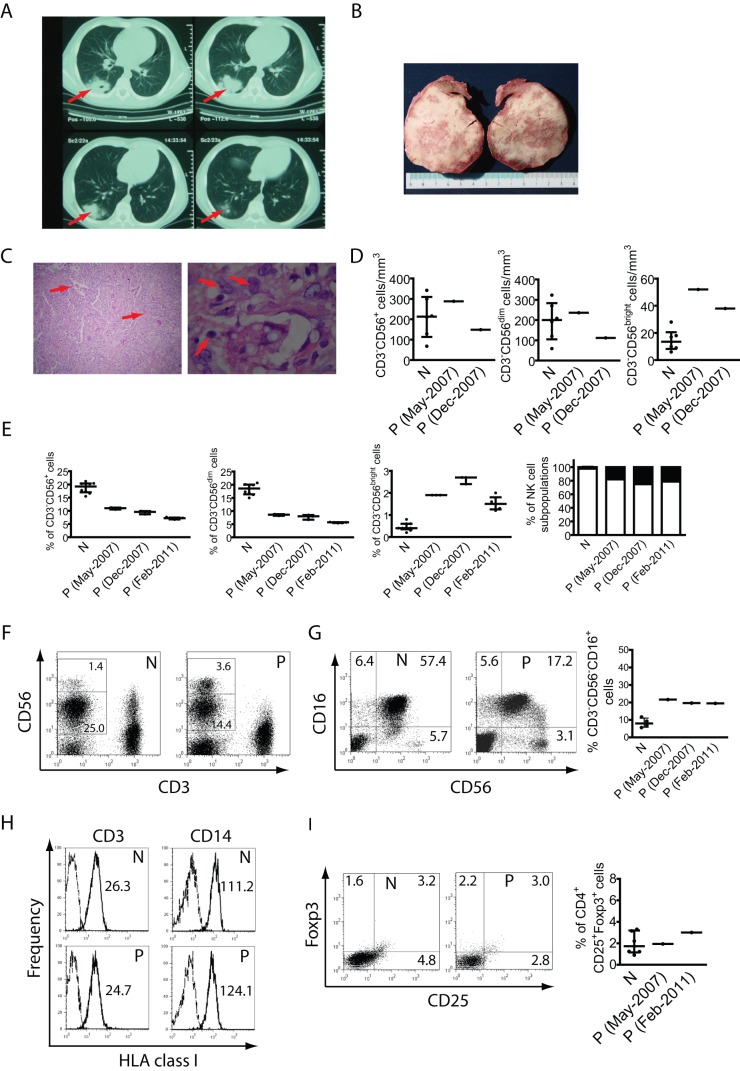
Lung infection and analysis of blood mononuclear cells. **A**) CT scan of the lungs of the patient, showing an area of consolidation of 43×60 mm in right lower lobe parenchyma (indicated by arrows). **B**) Lung biopsy (sliced in two pieces) with visible cavitated lesions with mucoid material. **C**) Hematoxylin/eosin staining of the lung biopsy showing the infiltration of inflammatory cells with predominance of histiocytes and multinucleated giant cells containing PAS-positive spherical bodies of 5 to 10 μm in diameter (indicated by arrows). The morphological characteristics of these PAS-positive spherical bodies are compatible with *Cryptococcus neoformans* infection. **D**) Absolute numbers of total NK cells, CD3^−^CD56^dim^ cells and CD3^−^CD56^bright^ cells in blood of 6 healthy normal donors (N) and in blood from two draws from different dates (indicated in parentheses) from the patient (P). **E**) PBMCs from 9 different normal donors (N) and from different blood draws from different dates (indicated in parentheses) from the patient (P) were stained with anti-CD3 and anti-CD56 mAbs, and the percentage of NK cells (CD3^−^CD56^+^ cells), CD3^−^CD56^dim^ and CD3^−^CD56^bright^ cells within the whole mononuclear cell population (PBMCs) were calculated and depicted as dot plots. Also, the relative abundance of CD3^−^CD56^bright^ and CD3^−^CD56^dim^ cells was calculated and depicted (CD3^−^CD56^bright^ are shown in black bars; CD3^−^CD56^dim^ cells are shown in white bars). Each sample of the patient was tested at least twice, and individual values obtained are shown as a dot in the graphs. Interquartile ranges (IQR) are indicated in the graphs of panels D and E. **F**) Representative dot plots of lymphoid cells gated according to their FSC and SSC parameters. The numbers in each region correspond to the percentage of CD3^−^CD56^dim^ and CD3^−^CD56^bright^ cells within the lymphoid population (gated according to their FSC and SSC parameters). **G**) Representative dot plots to show the percentage of CD3^−^CD56^−^CD16^+^ NK cells within the CD3^−^ lymphoid cell population in a healthy normal control (N) and in one sample of the patient (P). The numbers in each quadrant correspond to the percentage of CD16^+^CD56^−^, CD16^+^CD56^dim^ and CD16^−^CD56^bright^ NK cells. Right graph: data from 3 healthy normal controls and 3 blood samples from different dates from the patient (P). Percentages depicted correspond to the percentage of CD3^−^CD56^−^CD16^+^ cells within the total NK cell population (CD16^+^CD56^−^ + CD16^+^CD56^dim^ + CD16^−^CD56^bright^ cells). **H**) PBMCs from normal donors (N) and from the patient (P) were stained with anti-CD3, anti-CD14 and anti-HLA class I mAbs to assess HLA class I expression (continuous line) on T cells (CD3^+^ cells) and monocytes (CD14^+^ cells). Data presented correspond to representative histograms. Dashed lines: IC mAb. The numbers inserted in the graphs correspond to the MFI. **I**) PBMCs from normal donors (N) and from the patient (P) were stained with anti-CD4, anti-CD25 and anti-Foxp3 to assess the percentage of Tregs. The dot plots correspond to Foxp3 *vs*. CD25 in CD4^+^ cells gated from the FSC vs. SSC plots. The percentages in each quadrant are shown. The right graph shows the percentage of Tregs in PBMCs from 6 healthy normal controls and in two blood samples from different dates (indicated in parentheses) from the patient (P). One representative experiment from 3 independent analyses is shown in C and D. PBMCs from blood samples obtained in February 2011 were used for panels F, H and I.

*In vitro* evidence indicates that CD56^bright^ NK cells are precursors of CD56^dim^ NK cells. However, *in vivo* evidence about such lineage development is still lacking. As mouse NK cells are different from human NK cells in many aspects [1], results from knockout mice cannot be extrapolated to humans. Conversely, human NK cell deficiencies are helpful to unravel the immunobiology of NK cells but these conditions are very rare [9]. Absolute or functional NK cell deficiencies are associated with recurrent viral infections [9], confirming their role as effector cells against such pathogens but deficiencies that contribute to elucidate NK cell developmental pathways remain unknown. Here, we describe a human immunodeficiency-like condition characterized by a reduced frequency of CD3^−^C56^dim^ cells with lower percentages of terminally differentiated NK cells, and accumulation of CD3^−^CD56^bright^ cells in peripheral blood that exhibit altered activation in response to IL-2 and IL-15. Hence, our results contribute to further unravel human NK cell ontogeny as we provide strong evidence that support the idea that *in vivo* CD56^bright^ NK cells differentiate into CD56^dim^ NK cells.

**Table 1:** Laboratory findings in the patient along time.

Age (years)	13	14	16	17	Reference values
Serum IgG (mg/dl)	1190	1270	1130	1040	984–1544
Serum IgA (mg/dl)	290	247	112	144	112–252
Serum IgM (mg/dl)	196	80	100	91	82–220
Anti-TT Ab (UI/ml) *	0.5	0.8	ND †	ND	>0.1
Anti- pnemococcus Ab (mg/dl)	88	270	216	139	>113 ‡
Anti-measles virus Ab	+	ND	ND	ND	
Anti-HIV Ab	−	−	ND	ND	
Granulocyte percentage in whole blood	72	54	58	49	40–80%
Monocyte percentage in PBMCs (cells/mm^3^)	7 (168)	8 (156)	8 (208)	8 (557)	4–13%
T cell percentage in PBMCs (cells/mm^3^)					
CD3^+^	57 (1368)	66 (1289)	66 (1719)	58 (4041)	65–85%
CD4^+^	32 (768)	39 (729)	36 (936)	35 (2450)	36–46%
CD8^+^	19 (456)	23 (430)	25 (650)	20 (1400)	19–40%
B cell percentage in PBMCs (cells/mm^3^)					
CD20^+^	28 (672)	24 (467)	20 (520)	26 (1812)	7–23%
Proliferative response (cpm)					
PHA	40000	115000	ND	ND	74.000–135.000
ConA	79000	101000	ND	ND	54.000–104.000
Anti-CD3	46000	180000	ND	ND	55.000–91.000
PMA + Ionomycin	69000	275000	ND	ND	61.000–149.000
DTH	N §	ND	ND	ND	

* TT: Tetanus toxoid; †ND: not determined; ‡As established by the Argentinean Society of Pediatrics; §N: normal.

## Methods

Studies have been approved by the institutional review board and written informed consent was obtained from the parents of the patient (as he was not major at the beginning of these studies). In addition, written consent was obtained from the parents on the behalf of the minors involved in our study as well as from the major healthy volunteers that provided blood samples for this study. Also, all the participants (or the parents of the participants) of this study gave written consent for the publication of the clinical data gathered along this investigation.

**Figure 2:**
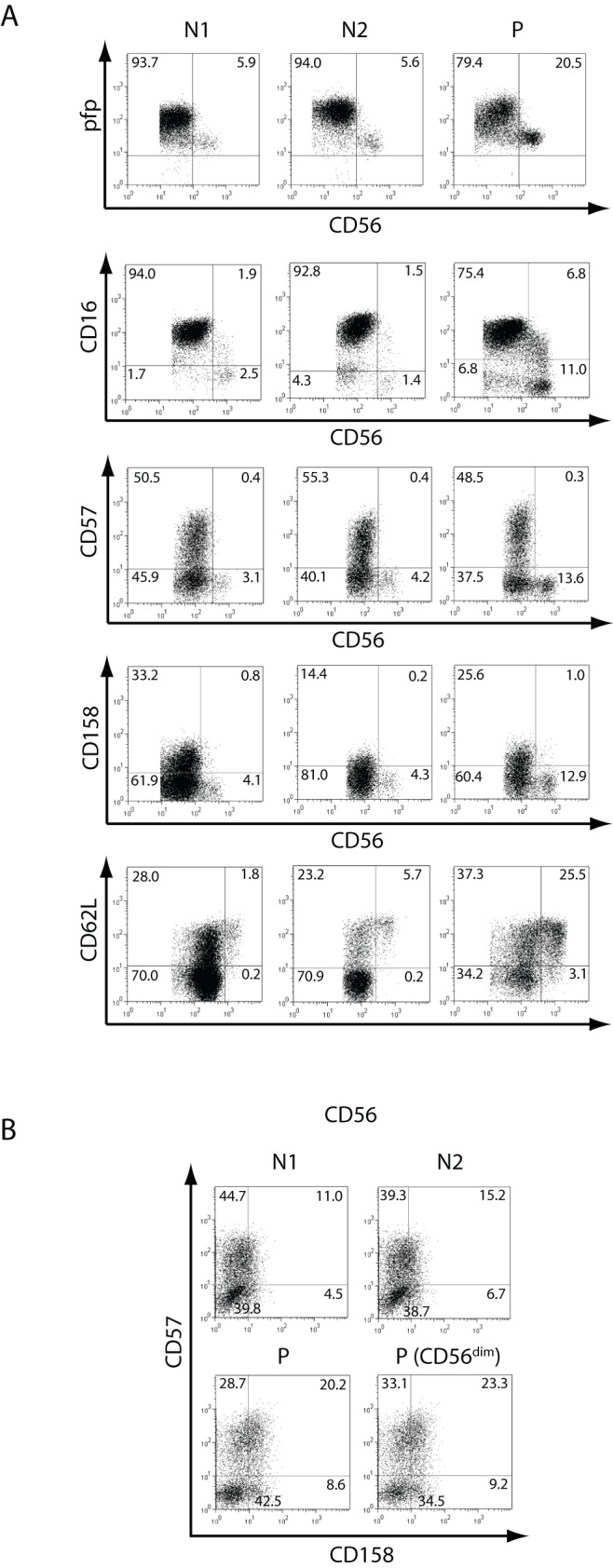
Phenotype of NK cells. **A**) PBMCs from two normal (healthy) donors (N1 and N2) and from the patient (P) were stained with anti-CD3 and anti-CD56, and anti-pfp, anti-CD16, anti-CD57, anti-CD158 (KIR) or anti-CD62L, and fluorescence derived from this third fluorochrome-labeled mAb was depicted against CD56 after gating NK cells as CD3^−^CD56^+^ as dot plots. **B**) Expression of CD57 and CD158 in NK cells (depicted as dot plots and gated as CD3^−^CD56^+^ cells) from N1, N2 and the patient (P), and on CD3^−^CD56^dim^ cells from P. The percentage in each quadrant is shown. Results correspond to one from 3 independent experiments. PBMCs from blood samples obtained in May 2007 were used.

### Antibodies, cytokines and reagents

Recombinant human IL-2, IL-12 and IL-15 were from PeproTech; recombinant human IL-18 was from MBL International. The following mAbs against human molecules were used: FITC-, PE- and SPRD-labeled anti-CD3 (UCHT-1, Southern Biotech); PE/Cy7-labeled anti-CD3 (UCHT-1, Biolegend); FITC-labeled anti-CD14 (HCD14 Biolegend); PE-labeled anti-HLA class I (W6/32, Biolegend); PE/Cy5-labeled anti-CD56 (N901, Beckman Coulter); FITC-labeled anti-CD16 (eBioCB16, eBioscience); FITC-labeled anti-CD57 (HCD57, Biolegend); FITC- or PE-labeled anti-CD62L (DREG-56, Southern Biotech); FITC-labeled anti-perforin (pfp, dG9, Biolegend); PE-labeled anti-IFN-γ (4S.B3, Biolegend); FITC-labeled anti-CD107a (1D4B, BD Biosciences); PE-labeled anti-CD25 (BC96, Biolegend); FITC-labeled anti-CD69 (FN50, BD); PE-labeled anti-NKG2D mAb (clone 1D11, Biolegend); PE-labeled anti-NKp46 (clone 9E2; Biolegend); FITC-labeled anti-CD226 (DNAM-1, clone DX11, BD Pharmingen); PE-labeled anti-CD244 (2B4, clone C1.7, Biolegend); PE-labeled anti CD94 (clone DX22; Biolegend); APC-labeled anti-NKG2A (clone 131411, R&D); mAb isotype-matched control mAb (IC, eBioscience). KIR analysis was performed with a mix of PE-labeled anti-CD158a, h (EB6.B) and the anti-CD158b1, b2 (GL183) mAbs (both from Beckman Coulter). For regulatory T cell staining, the Alexa Fluor 488, FoxP3, CD4, CD25 FoxP3 staining kit from BD was used. 7AAD Viability Staining Solution was from Biolegend.

**Figure 3:**
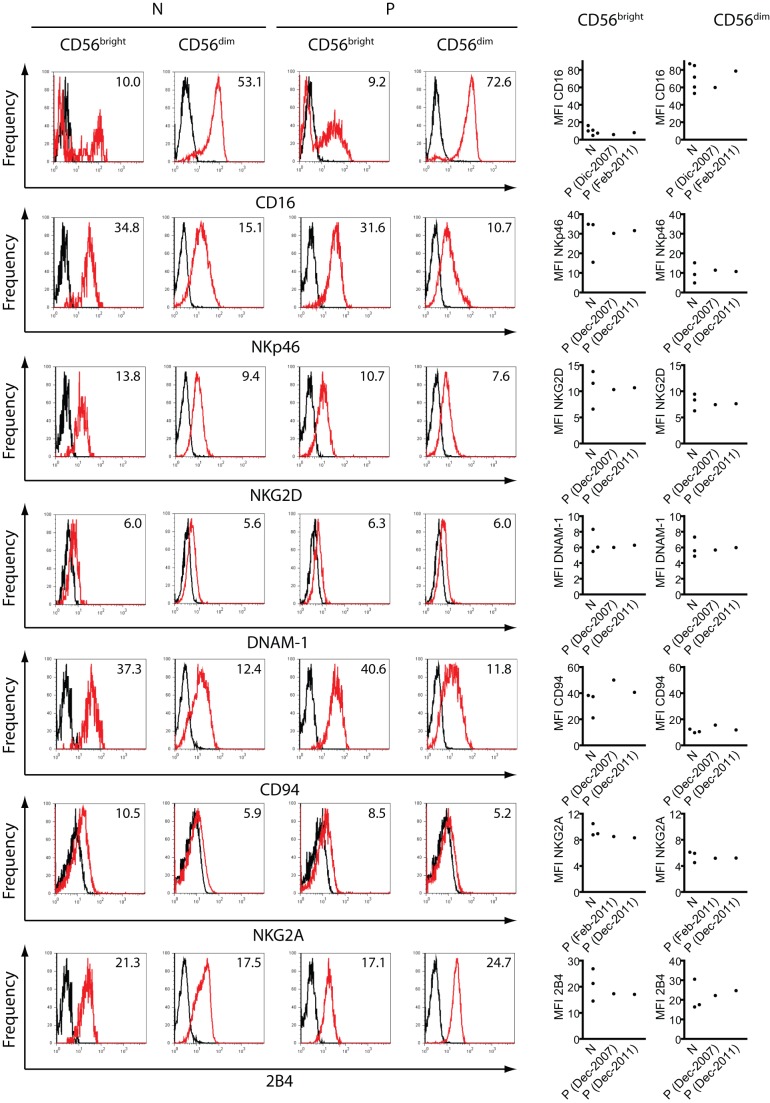
Expression of NK cell receptors. PBMCs from at least 3 different normal(healthy) donors (N) and from different blood draws from different dates (indicated in parentheses) from the patient (P) were stained with anti-CD3, anti-CD56, and anti-CD16, anti-NKp46, anti-NKG2D, anti-DNAM-1, anti-CD94, anti-NKG2A or anti-2B4 as described in Methods. Fluorescence derived from the third fluorochrome-labeled mAb on CD3^−^CD56^bright^ or CD3^−^CD56^dim^ cells was depicted. Representative histograms are shown on the left with the corresponding MFI inserted in the histrogam, while the MFI for each receptor of the healthy normal donors and the 2 different samples of the patient are depicted on the right graphs. PBMCs from blood samples obtained in December 2011 were used.

**Figure 4:**
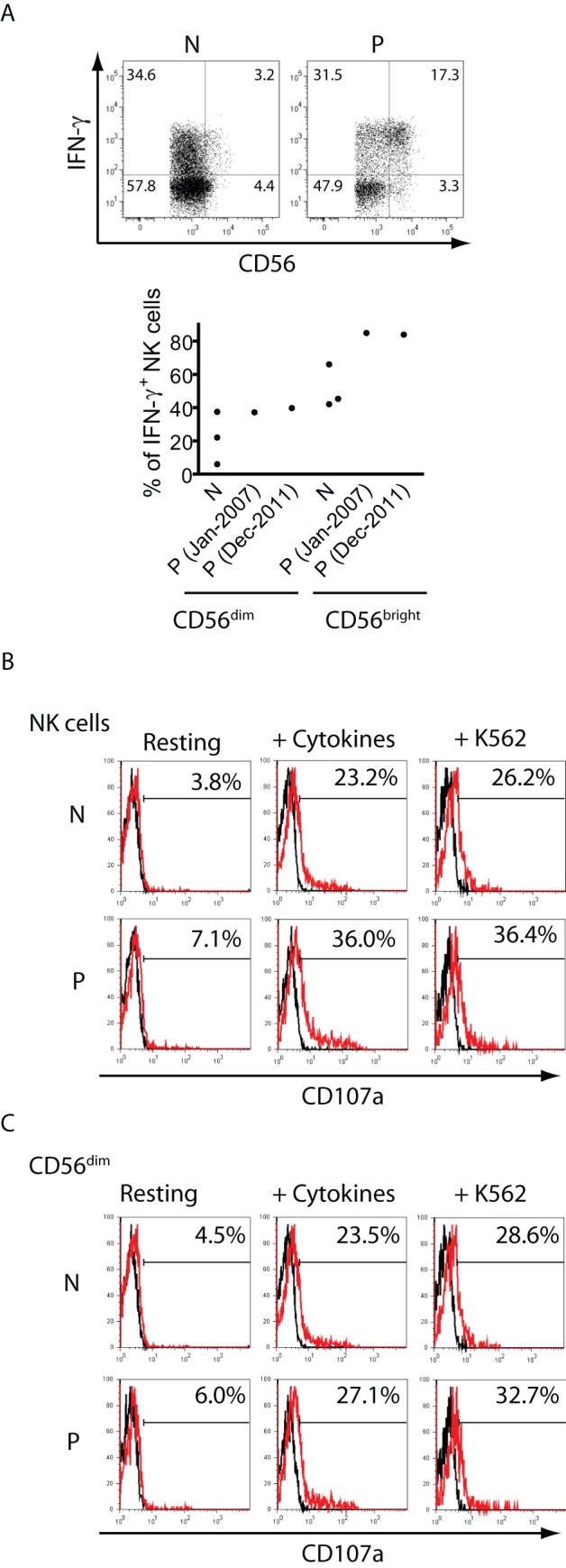
Functional response of NK cells. **A**) NK cells from a normal (healthy) donor (N) or from the patient (P) were stimulated overnight with IL-12, IL-15 and IL-18, and the percentage of IFN-γ-producing NK cells was assessed by FC. Upper panels: Dot plots presented correspond to CD3^−^CD56^+^ gated cells from one healthy normal donor and from one sample of the patient. Lower graph: individual data from three healthy normal donors and from two different samples of the patient. **B, C**) NK cells from a healthy normal donor (N) or from the patient (P) were cultured overnight in the absence (Resting) or in the presence of IL-12, IL-15 and IL-18 (+ Cytokines) or K562 cells at an 1∶1 ratio (+ K562), and cell surface CD107a (red histograms) was assessed on gated CD3^−^CD56^+^ cells (**B**) or on gated CD3^−^CD56^dim^ cells (**C**) by FC and depicted as histograms. The numbers in the histograms indicate the percentage of CD107a^+^ cells. Markers were set with cells stained with the IC mAb (black histograms) to leave less than 1% of positive cells. Experiments were repeated twice with NK cells from two different healthy donors and two different blood draws from the patient with similar results. PBMCs from blood samples obtained in May 2007 were used for panels B and C.

**Table 2:** Expression of NK cell markers in CD56dim and CD56bright cells from two healthy normal donors (N1, N2) and from the patient (P).

	CD56^dim^								
Marker	N1 (resting)	N2 (resting)	P (resting)	N1 (+ IL-2)	N2 (+ IL-2)	P (+ IL-2)	N1 (+ IL-15)	N2 (+ IL-15)	P (+ IL-15)
pfp	100	100	100	100	100	100	100	100	100
CD16	98.2	95.6	91.7	90.8	93.0	63.1	92.1	87.8	70.4
CD57	52.4	58.0	56.4	58.8	56.6	43.9	62.0	62.3	49.9
CD158	34.9	15.1	29.8	71.0	63.6	50.1	73.3	65.2	50.6
CD62L	28.6	24.7	52.2	16.9	19.3	12.4	ND	ND	ND

	CD56^bright^								
Marker	N1 (resting)	N2 (resting)	P (resting)	N1 (+ IL-2)	N2 (+ IL-2)	P (+ IL-2)	N1 (+ IL-15)	N2 (+ IL-15)	P (+ IL-15)
pfp	100	100	100	100	100	100	100	100	100
CD16	43.2	51.7	38.2	78.5	77.3	23.8	86.1	83.2	28.4
CD57	11.4	8.7	2.2	59.7	54.1	10.0	69.5	68.0	15.1
CD158	16.3	4.4	7.2	54.7	21.3	27.9	61.3	35.0	27.5
CD62L	90.0	96.6	89.2	26.9	32.1	53.5	ND	ND	ND

Numbers correspond to the percentage of cells expressing the indicated marker.

### PBMCs and NK cells

Human peripheral blood mononuclear cells (PBMCs) and NK cells were isolated from blood of healthy volunteers or from the patient described in this work (called “P”) using Ficoll-Paque™ Plus (Amersham Biosciences) centrifugation and the RosetteSep kit (StemCell), respectively. During the initial testing, we recruited age-matched healthy donors to compare the patient with these age-matched samples (hematological data, percentage of NK cells and CD56^dim^ and C56^bright^ populations). However, as the values of this age-matched population was similar to the values for adult population, and considering that the patient reached the age of 17 at the end of this study, in subsequent (retroactive) experiments we used healthy donors that correspond to young adults (ages between 25 and 30). Purity of isolated NK cells (CD3^−^CD56^+^ cells) was above 90%, as assessed by flow cytometry (FC). In some experiments, PBMCs were cultured with IL-2 (0.8 ng/ml) or IL-15 (10 ng/ml) in RPMI 1640 (Sigma) supplemented with 10% fetal bovine serum (NatoCor, Córdoba, Argentina), sodium pyruvate, glutamine and gentamicin (Sigma) for up to 5 days.

**Figure 5:**
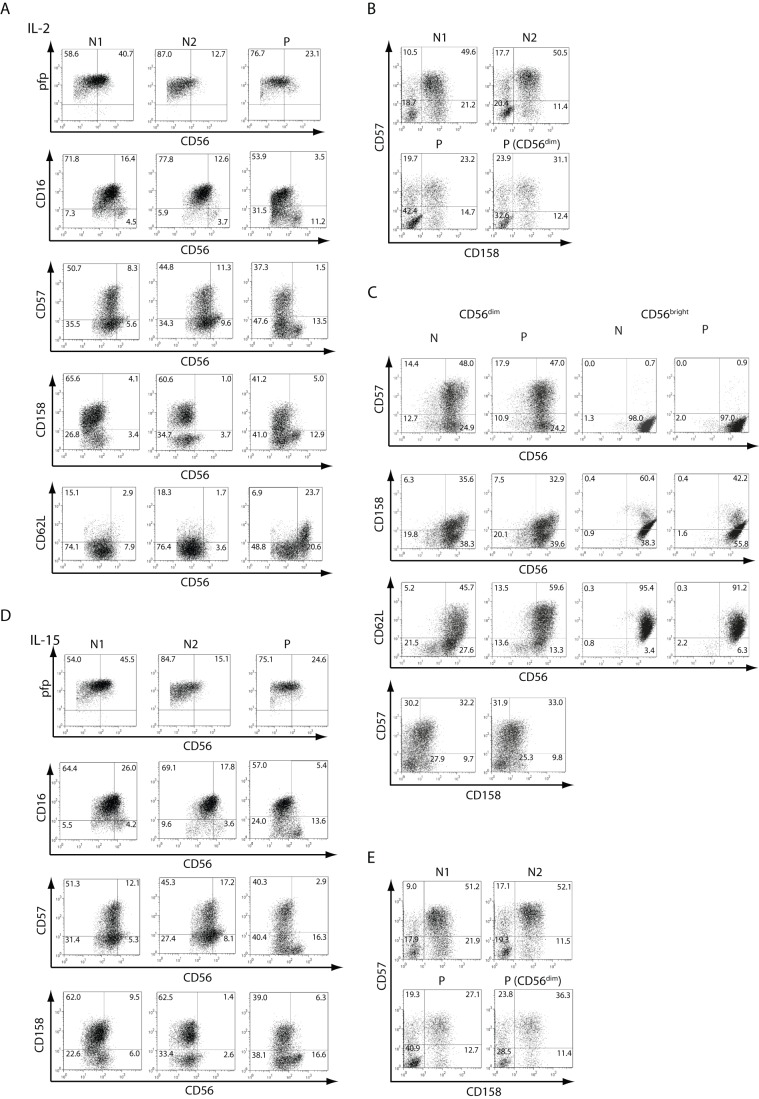
Phenotype of NK cells stimulated with IL-2 or IL-15. PBMCs from two healthy normal donors (N1 and N2) and from the patient (P) (**A**, **B**) or sorted CD56^dim^ and CD56^bright^ NK cells from one healthy normal donor and from the patient (**C**) were stimulated for 3 days with IL-2. Also, PBMCs from two healthy normal donors (N1 and N2) and from the patient (P) were stimulated for 3 days with IL-15 (**D, E**). Thereafter, cells were harvested and stained with anti-CD3 and anti-CD56, and a third fluorochrome-labeled mAb (indicated on the *y*-axis). Also, expression of CD57 and CD158 in NK cells (gated as CD3^−^CD56^+^ cells) from N1, N2 and the patient (P), and on CD3^−^CD56^dim^ cells from P was plotted for cells stimulated with IL-2 or IL-15 (**B, E**). Expression of CD57 and CD158 on sorted and IL-2-stimulated CD56^dim^ NK cells from one healthy normal donor and from the patient is also shown (**C**). Data are depicted as dot plots. The percentages in each quadrant are shown. Results correspond to one from 3 independent experiments.

### Flow cytometry and cell sorting

Cells were stained with specific fluorochrome-labeled mAbs, analyzed in a FACSAria flow cytometer (BD), and data were processed with the FlowJo software (Tree Star Inc., Ashland, OR). Numerical data presented are the percentage of cells in the quadrants in the dot plots or to the geometric mean fluorescence intensity (MFI) in the histograms. Also, CD3^−^CD56^dim^ and CD3^−^CD56^bright^ cells were sorted with the FACSAria cell sorter up to >99% of purity.

**Figure 6:**
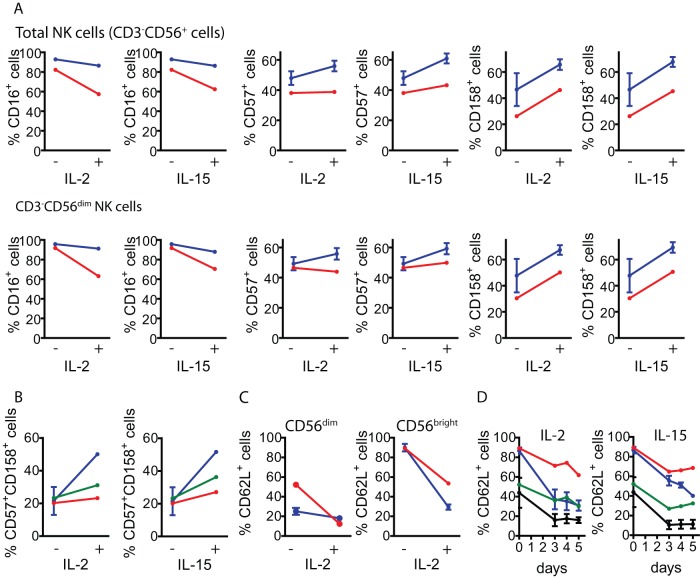
Percentage of CD16^+^, CD57^+^, CD158^+^ and CD62L^+^ NK cells before and after stimulation with IL-2 or IL-15. **A**) Percentage of CD16^+^, CD57^+^ and CD158^+^ cells before (−) and after stimulation of PBMCs with IL-2 or IL-15 (+) in total NK cells (CD3^−^CD56^+^ cells, upper line of graphs) and in CD56^dim^ NK cells (lower lane of graphs) from healthy normal donors (blue) and from the patient (red). **B**) Percentage of CD57^+^CD158^+^ cells before (−) and after stimulation of PBMCs with IL-2 or IL-15 (+) in total NK cells (CD3^−^CD56^+^ cells) and in CD56^dim^ NK cells from the same samples. Blue: NK cells from healthy donors; red: NK cells from the patient; green: CD3^−^CD56^dim^ NK cells from the patient. **C**) Percentage of CD62L^+^ cells before (−) and after stimulation of PBMCs with IL-2 (+) in CD3^−^CD56^dim^ cells and in CD3^−^CD56^bright^ cells from the same samples. Blue: CD3^−^CD56^+^ cells from healthy donors; red: CD3^−^CD56^+^ cells from the patient; green: CD3^−^CD56^dim^ cells from the patient. **D**) Percentage of CD62L^+^ cells before (0) and after 3 to 5 days of stimulation of PBMCs with IL-2 (left graph) or IL-15 (right graph) in CD3^−^CD56^dim^ and CD3^−^CD56^bright^ NK cells from healthy donors and from the patient. Black: CD3^−^CD56^dim^ cells from healthy donors; green: CD3^−^CD56^dim^ cells from the patient; blue: CD3^−^CD56^bright^ cells from healthy donors; red: CD3^−^CD56^bright^ cells from the patient. Six healthy donors samples were used for analysis of CD16 and CD57; two healthy donor samples were used for analysis of CD158, CD57 and CD62L expression. One representative experiment from 3 independent analyses is shown. PBMCs from blood samples obtained in December 2007 were used.

### IFN-γ production by NK cells

PBMCs from normal donors or from the patient were stimulated overnight at 37°C with IL-12 (10 ng/ml), IL-15 (1 ng/ml) and IL-18 (10 ng/ml). During the last 4 h of culture, GolgiStop and GolgiPlug reagents (BD) were added, following the instructions provided by the manufacturer. Thereafter, cells were stained with anti-CD3 and anti-CD56 mAbs, permeabilized with Cytofix/Cytoperm (BD) and stained with the anti-IFN-γ mAb to assess IFN-γ producing cells within the CD3^−^CD56^dim^ and CD3^−^CD56^bright^ NK cell subpopulations by FC.

### Degranulation of NK cells

NK cells were cultured overnight at 37°C with IL-12, IL-15 and IL-18 or K562 target cells. During the last 4 h, the anti-CD107a or an IC mAbs were added together with Golgi-Plug and Golgi-Stop (BD). Thereafter, cells were stained with anti-CD3 and anti-CD56 mAbs, and the percentage of CD107a^+^ cells within the CD3^−^CD56^+^ or within the CD3^−^CD56^dim^ cell populations was determined.

### Statistical analysis

A paired *t*-Student test was used to compare expression of NK cell receptors between CD3^−^CD56^dim^ and CD3^−^CD56^bright^ cells.

## Results

### NK cell number and phenotype in blood of a human patient with melanoma and opportunistic fungal infection

We studied a case of a pediatric patient (P) that presented repeated upper airway infections and that at the age of 12 developed an ulcerative spitzoid melanoma in his right ear. Although the melanoma was successfully removed by surgery, one year later the patient presented pneumonia in the lower right lobe, accompanied by fever and bloody stools. A computed tomography (CT) of the chest revealed the presence of an opaque mass (Fig. 1A) for which a lung biopsy was taken (Fig. 1B). The analysis of this biopsy revealed the presence of *Cryptococcus neoformans* (Fig. 1C). The fungal infection was non-invasive, there was no evidence about other infectious agent in the lung to suspect that the cryptococcosis could have been a super-infection or a case of commensalism, and patient was successfully treated with fluconazol. Nonetheless, the fact that within one year the patient suffered from a melanoma and a deep fungal infection in the lung led to the suspicion that the patient might be experiencing some sort of immunodeficiency. Therefore, further immunological studies were performed. Standard laboratory tests (Table 1) ruled out classical primary or secondary immunodeficiencies (this patient was HIV-negative). Also, subclasses of IgG (IgG1, IgG2, IgG3 and IgG4), antibody activity and complement activity were normal. The patient was Purified Protein Derivative from *Mycobacterium tuberculosis* (PPD)-negative and had a complete vaccination schedule with no adverse reactions. Although the patient did not show further medical problems, periodic blood examination covering a period of 4 years revealed a slight decrease in the percentage of CD3^+^ cells within PBMCs at the age of 13 and 17, accompanied by reduced proliferative response against PHA or anti-CD3 stimulation, absolute numbers of NK cells that were within the range of healthy individuals but an altered frequency of CD3^−^C56^dim^ and CD3^−^C56^bright^ NK cells in peripheral blood when compared to healthy normal controls (Fig. 1D, *left graph* and Fig. 1E). However, only the altered frequency of NK cells was stable and consistent along time. Therefore, we centered our efforts in a deeper analysis of this cell compartment. This reduced frequency of peripheral blood NK cells was mostly due to the presence of reduced percentages of CD56^dim^ NK cells (Fig. 1D and 1E) accompanied by increased percentages and absolute numbers (cells/mm^3^) of CD56^bright^ NK cells in PBMCs (Fig. 1D–F). These results indicate that this patient exhibits a stable phenotype characterized by an accumulation of CD3^−^CD56^bright^ cells accompanied by a reduction in CD3^−^CD56^dim^ cells in peripheral blood and suggest that there could be a blockade in the transition of CD3^−^CD56^bright^ cells to CD3^−^CD56^dim^ cells *in vivo*. A more detailed analysis revealed that the patient also seems to exhibit an accumulation of CD3^−^CD56^−^CD16^+^ NK cells (Fig. 1G).

Accumulation of CD56^bright^ NK cells has been observed in certain TAP deficiencies and after treatment with Daclizumab, a humanized anti-CD25 mAb [10]. TAP deficiencies cause reduced expression of HLA class I molecules, but unaltered amounts of HLA class I molecules were detected on T cells and monocytes of this patient (Fig. 1H). Daclizumab interferes with the IL-2 signaling, which is a pathway necessary to generate regulatory T cells (Treg, CD4^+^CD25^+^Foxp3^+^ cells). However, this patient exhibited percentages of CD4^+^CD25^+^Foxp3^+^ cells in peripheral blood that were similar to healthy normal donors (Fig. 1I). Thus, this patient does not seem to exhibit a TAP deficiency and it is unlikely that he exhibits a blockade in the IL-2 signaling pathway.

To further characterize NK cells from this patient, we performed phenotypic and functional studies. A phenotypic analysis demonstrated percentages of perforin^+^, CD16^+^, CD57^+^, CD158^+^ and CD62L^+^ cells within the CD56^dim^ and CD56^bright^ cell populations similar to healthy donors, as well as slightly reduced percentages of CD57^+^CD158^+^ cells within the total NK cell population and also within gated CD3^−^CD56^dim^ NK cells, when compared to healthy controls (Fig. 2 and Table 2). In addition, we analyzed the expression of a large panel of NK cell receptors on CD56^bright^ and CD56^dim^ NK cells (Fig. 3). We observed higher amounts of CD16 on CD56^dim^ cells than on CD56^bright^ cells from both, healthy controls and the patient, and these differences were statistically significant (expression on CD56^dim^ vs. CD56^bright^ NK cells: *p*<0.01 for healthy donors and *p*<0.001 for the patient). Conversely, we observed higher amounts of NKp46, NKG2D, CD94 and NKG2A on CD56^bright^ cells than on CD56^dim^ cells from both, healthy controls and the patient. The higher expression of NKp46, NKG2D, CD94 and NKG2A on CD56^bright^ NK cells than on CD56^dim^ cells were statistically significant for each receptor and for NK cells from healthy donors or from the patient (*p*<0.05 in all cases). Conversely, the expression of DNAM-1 and 2B4 were similar on CD56^bright^ NK cells and CD56^dim^ cells from healthy donors or from the patient. Besides the different expression of CD16, NKp46, NKG2D, CD94 and NKG2A on CD56^bright^ *vs*. CD56^dim^ cells, no differences were observed in their expression between CD56^bright^ NK cells from the patient when compared to CD56^bright^ NK cells from healthy donors, and between CD56^dim^ NK cells from the patient when compared to CD56^dim^ NK cells from healthy donors. In addition, functional analysis revealed that CD3^−^CD56^dim^ and CD3^−^CD56^bright^ cells of this patient produced IFN-γ in response to IL-12, IL-15 and IL-18 (Fig. 4A). Although it appeared that CD56^bright^ cells from the patient produced more IFN-γ than the healthy controls, the sample size was not enough to decide whether this difference was statistically significant. Besides, these results indicate that the patient's NK cells do not exhibit a defect in the capacity to produce IFN-γ. Moreover, NK cells and CD56^dim^ NK cells of the patient also did not exhibit an alteration in the degranulation capacity (expression of cell surface CD107a) after stimulation of NK cells with IL-12, IL-15 and IL-18 or after co-culture with K562 cells (Fig. 4B).

These results suggest that this patient exhibits an altered frequency of NK cells, an increase in the absolute numbers of CD56^bright^ NK cells in blood, and CD56^dim^ NK cells that exhibit a slightly reduced percentage of terminally differentiated cells (CD57^+^CD158^+^) but with unaltered expression of the main receptors involved in NK cell activation (CD16, NKp46, NKG2D and DNAM-1) and regulation (CD94/NKG2A), and in their IFN-γ production and degranulation in response to cytokines or K562 cells.

### Inappropriate *in vitro* maturation/activation of NK cells in response to cytokines supports *in vivo* differentiation of CD56^bright^ cells into CD56^dim^ cells

Next, PBMCs of the patient were stimulated with IL-2 or IL-15 and expression of the same cell surface markers as in resting NK cells was assessed (Fig. 5 and Table 2). We observed that upon stimulation with IL-2 or with IL-15, CD56^bright^ NK cells from the patient up-regulated expression of perforin (Fig. 5), CD25 (the α chain of the high affinity receptor of IL-2, *not shown*), and CD69 (*not shown*) in a similar manner as NK cells from healthy donors. However, upon stimulation with these cytokines, CD56^bright^ NK cells of the patient remained clearly distinguishable from CD56^dim^ NK cells in the dot plots (compare Fig. 2 to Fig. 5B and Fig. 5D). Conversely, it was hard to distinguish the CD56^bright^ NK cells from the CD56^dim^ NK cells in healthy donors, appearing both as only one CD56^+^ homogeneous cell population.

Moreover, less CD56^dim^ NK cells from the patient, compared to healthy controls, acquired CD57 and CD158 upon stimulation with IL-2 or IL-15 (Fig. 5A and 5B, and Fig. 6A). Also, a substantial fraction of CD56^dim^ cells showed a reduction in the percentage of CD16^+^ cells (Fig. 6A and Table 2), and less total NK cells and CD56^dim^ cells from the patient, compared to healthy controls, became CD57^+^CD158^+^ upon stimulation with IL-2 or IL-15 (Fig. 5B, Fig. 5E and Fig. 6B). Overall, these results suggest that in the patient, less NK cells could progress to an activated phenotype and acquire a terminally differentiated phenotype in response to IL-2 or IL-15. In addition, we observed that CD3^−^CD56^bright^ cells of the patient exhibited a defective down-regulation of CD62L upon stimulation with IL-2 (Fig. 5A, Fig. 6C and Table 2) that was not due to a slower kinetic of CD62L down-regulation as even at prolonged stimulation periods with IL-2 or IL-15, more than 60% of CD3^−^CD56^bright^ NK cells of the patient while less than 20% of CD3^−^CD56^bright^ cells from healthy controls remained CD62L^+^ (Fig. 6D). Of note, the inability to down-regulate CD62L and the sustained high expression of CD56 was not due to apoptosis induction as CD56^bright^ NK cells remained viable after 5 days of stimulation with IL-15 (*not shown*). Experiments performed with sorted CD56^dim^ and CD56^bright^ NK cells stimulated with IL-2 revealed that both NK cell subpopulations (from the patient and from the healthy control that was simultaneously analyzed) up-regulated CD56 expression (Fig. 5C). However, sorted CD56^dim^ cells from healthy controls and from the patient did not up-regulate CD158 in response to IL-2, while sorted CD56^dim^ and CD56^bright^ cells did not down-regulate expression of CD62L. These results indicate that IL-2 alone cannot provide sufficient maturation signals to sorted NK cell subpopulations.

In summary, the results presented suggest that this patient exhibits a clinical picture that is compatible with a novel immunodeficiency-like condition characterized by *a*) low frequency of CD56^dim^ NK cells in peripheral blood that cannot properly acquire expression of, CD57 and CD158, and maintain expression of CD16 upon stimulation with IL-2 or IL-15; and *b*) an increase in the frequency and absolute numbers of CD56^bright^ NK cells that do not down-regulate CD62L upon stimulation with these cytokines. Overall, these data might suggest the existence of a defect that compromises NK cell maturation and activation and support the idea that *in vivo* CD3^−^CD56^bright^ cells differentiate into CD3^−^CD56^dim^ NK cells.

## Discussion

NK cells are pivotal players of the immune response against tumors and viruses in humans and mice. Although much information about NK cell lineage development has been obtained using genetically modified animals, information about human NK cell ontogeny is limited mostly due to the scarcity of human NK cell deficiencies. Some data have emerged from studies performed in blood of patients after bone marrow transplantation [11]. Also, four NK cell developmental intermediates have been indentified in human lymph nodes and tonsils [12]. However, an open and important question is whether CD56^dim^ and CD56^bright^ NK cells belong to separate lineages of whether CD56^bright^ NK cells are immature precursors of CD56^dim^ NK cells. *In vitro* evidence favors the second possibility [10], [13]–[18], but *in vivo* evidence is still lacking. Here, we describe the first human immunodeficiency-like condition characterized by low frequency of CD56^dim^ NK cells with an overt and persistent increased frequency and absolute numbers of CD56^bright^ NK cells in peripheral blood. This particular phenotype was accompanied by a slight increased frequency of CD3^−^CD56^−^CD16^+^ NK cells, which are expanded during chronic viral infections, are thought to be a source of chemokines relevant for anti-viral immunity, and tentatively derive from CD56^dim^ NK cells [19].

Increased percentage of CD56^bright^ NK cells with normal numbers of total NK cells have been observed in different physiological and pathological conditions [20]–[24], but none of them seem to be plausible in the patient herein studied. Recently, two reports described mutations in the minichromosome maintenance complex component 4 (MCM4) that caused NK cell deficiency and adrenal insufficiency [25], [26]. In one report, reduced NK cell numbers were observed but their phenotype and function was not investigated [26]. In the other report it was observed that the patients that carry the mutation exhibited reduced amounts of CD56^dim^ NK cells and lower percentages of pfp^+^, CD57^+^ and CD94^med/low/−^ NK cells in blood, and it was concluded that CD56^bright^ NK cells are largely depend on MCM4 to proliferate and generate C56^dim^ NK cells [25]. However, normal amounts of CD56^bright^ NK cells in blood of the patients were detected. Conversely, in the patient that we present in this work, a consistent increase in absolute counts of CD56^bright^ NK cells was detected and he did not exhibit a growth retardation or adrenal insufficiency.

A detailed phenotypic analysis of NK cells from our patient revealed percentages of perforin^+^, CD16^+^, CD57^+^, CD158^+^ within the CD56^dim^ subpopulation, and CD62L^+^ cells within the CD56^bright^ subpopulation that were similar to healthy donors. However, the patient exhibited slightly decreased percentages of CD57^+^CD158^+^ CD56^dim^ NK cells. Expression of CD57 and CD158 has been associated with the acquisition of a terminally differentiated phenotype by NK cells [27], [28], [29] for which it appears that the patient presents less terminally differentiated NK cells in peripheral blood. Also, CD56^dim^ and CD56^bright^ NK cell subpopulations expressed unaltered amounts of the main receptors involved in NK cell activation (CD16, NKp46, NKG2D and DNAM-1) and regulation (CD94/NKG2A), and did not exhibit a defect in IFN-γ production upon stimulation with IL-12, IL-15 and IL-18. Also, CD3^−^CD56^dim^ NK cells did not present an altered degranulation in response to cytokines and K562 cells. Since we observed a decrease in CD56^dim^ NK cell frequency with a concomitant increase in the frequency and absolute numbers of CD56^bright^ NK cells in peripheral blood, we reasoned that the patient may course with a mild immunodeficiency that affects NK cell maturation of CD56^bright^ cells and, eventually, activation of CD56^dim^ cells. Surprisingly, stimulation of PBMCs of this patient with IL-2 or IL-15 led to three major observations. First, the CD56^bright^ NK cell subpopulation remained clearly visible and distinguishable from the CD56^dim^ NK cell subpopulation. Second, these CD56^bright^ NK cells could not down-regulate CD62L upon stimulation with IL-2 or IL-15 but remained viable. And third, only a small fraction of CD56^dim^ NK cells acquired CD57 and CD158 upon stimulation with IL-2 or IL-15, while a reduction in the percentage of CD56^dim^CD16^+^ was observed. Although this difference indicates that some results obtained *in vitro* with cytokine-stimulated NK cells may not reflect an *in vivo* situation, our results indicate that CD56^bright^ cells of this patient could not progress along the normal pathway of maturation and that part of the CD56^dim^ NK cell pool of the patient could not acquire a terminally differentiated phenotype characterized by up-regulated expression of CD16, CD57 and CD158 [28], [29], [30]. The unusual behavior of the patient's NK cells was observed when IL-2 stimulations were performed using whole PBMCs. When sorted CD3^−^CD56^dim^ and CD3^−^CD56^bright^ cells were stimulated, we were unable to observe up-regulation of CD158 on CD56^dim^ cells and down-regulation of CD62L on CD56^bright^ from the patient and from the healthy control, suggesting that NK cells require more complex signals than IL-2 alone to trigger differentiation and maturation.

Previous reports [31], [32], [33] described the existence of a small subpopulation of CD56^bright^ NK cells characterized by the expression of CD16 (CD56^bright^CD16^+^) that would be a maturation intermediate. We may speculate that the blockade in the differentiation of CD56^bright^ cells into CD56^dim^ cells in the patient affects a fraction of the CD56^bright^CD16^−^ cells during their transition to CD56^bright^CD16^+^ cells because the former cells remain detectable upon stimulation with IL-2 or IL-15. On the other hand, down-regulation of CD62L and CD16 have been associated with the secretion and proteolytic activity of matrix metalloproteinases [34], [35]. As only CD56^bright^ NK cells but not CD56^dim^ NK cells exhibited defective down-regulated CD62L, it is very unlikely that the patient may display a defect in these enzymes. CD62L directs the traffic of CD56^bright^ cells to lymph nodes, where they down-regulate CD62L upon activation, facilitating the migration of NK cells to inflamed tissues. As CD56^bright^ NK cells from the patient failed to down-regulate CD62L upon stimulation, we suggest that they probably may traffic normally into lymph nodes but once they sense activating stimuli, they may display an altered trafficking pattern due to such faulty down-regulation of CD62L. This situation may lead to a weakened immunosurveillance of peripheral tissues exposed to environmental pathogens (such as skin and mucosa), which could have contributed to the development of a melanoma and the opportunistic fungal infections at these sites.

Of note, as the altered NK cell phenotype and response to cytokines described is partial and a fraction of NK cells from the patient achieved terminal differentiation, we suggest that he carries some sort of mild immunodeficiency or immunodeficiency-like condition that is probably the result of a heterozygous genotype that generates a “bottleneck” (instead of a complete blockade) that precludes CD56^dim^ NK cell development and activation at rates similar to healthy individuals. Such putative heterozygous genotype would preclude normal differentiation of CD56^bright^ cells to CD56^dim^ cells and in the acquisition of a CD62L^low/−^CD16^+^CD57^+^CD158^+^ phenotype by CD56^dim^ cells but would allow a sufficient number of NK cells to mature adequately and respond to activation stimuli, which in turn would provide a sufficient degree of NK cell activity to protect the patient from suffering major infections, in particular viral infections that are characteristic in patients with absolute or severe functional defects in NK cells [9]. Despite these speculations, our results provide evidence that indicates that *in vivo*, CD56^bright^ cells differentiate into CD56^dim^ NK cells, which constitute a contribution to a better understanding of NK cells ontogeny in humans.

## References

[1] Ferlazzo G, Pack M, Thomas D, Paludan C, Schmid D, et al (2004) Distinct roles of IL-12 and IL-15 in human natural killer cell activation by dendritic cells from secondary lymphoid organs. Proc Natl Acad Sci U S A 101: 16606–16611. 15536127 10.1073/pnas.0407522101 PMC534504

[2] Fernandez NC, Lozier A, Flament C, Ricciardi-Castagnoli P, Bellet D, et al (1999) Dendritic cells directly trigger NK cell functions: cross-talk relevant in innate anti-tumor immune responses in vivo. Nat Med 5: 405–411. 10202929 10.1038/7403

[3] Zwirner NW, Domaica CI (2010) Cytokine regulation of natural killer cell effector functions. Biofactors 36: 274–288. 20623510 10.1002/biof.107

[4] Lanier LL (2008) Up on the tightrope: natural killer cell activation and inhibition. Nat Immunol 9: 495–502. 18425106 10.1038/ni1581 PMC2669298

[5] Moretta A, Marcenaro E, Parolini S, Ferlazzo G, Moretta L (2008) NK cells at the interface between innate and adaptive immunity. Cell Death Differ 15: 226–233. 17541426 10.1038/sj.cdd.4402170

[6] Caligiuri MA (2008) Human natural killer cells. Blood 112: 461–469. 18650461 10.1182/blood-2007-09-077438 PMC2481557

[7] Strowig T, Brilot F, Munz C (2008) Noncytotoxic functions of NK cells: direct pathogen restriction and assistance to adaptive immunity. J Immunol 180: 7785–7791. 18523242 10.4049/jimmunol.180.12.7785 PMC2575662

[8] Frey M, Packianathan NB, Fehniger TA, Ross ME, Wang WC, et al (1998) Differential expression and function of L-selectin on CD56bright and CD56dim natural killer cell subsets. J Immunol 161: 400–408. 9647249

[9] Orange JS (2006) Human natural killer cell deficiencies. Curr Opin Allergy Clin Immunol 6: 399–409. 17088643 10.1097/ACI.0b013e3280106b65

[10] Poli A, Michel T, Theresine M, Andres E, Hentges F, et al (2009) CD56bright natural killer (NK) cells: an important NK cell subset. Immunology 126: 458–465. 19278419 10.1111/j.1365-2567.2008.03027.x PMC2673358

[11] Vitale C, Chiossone L, Morreale G, Lanino E, Cottalasso F, et al (2005) Human natural killer cells undergoing in vivo differentiation after allogeneic bone marrow transplantation: analysis of the surface expression and function of activating NK receptors. Mol Immunol 42: 405–411. 15607791 10.1016/j.molimm.2004.07.019

[12] Freud AG, Yokohama A, Becknell B, Lee MT, Mao HC, et al (2006) Evidence for discrete stages of human natural killer cell differentiation in vivo. J Exp Med 203: 1033–1043. 16606675 10.1084/jem.20052507 PMC2118285

[13] Chan A, Hong DL, Atzberger A, Kollnberger S, Filer AD, et al (2007) CD56bright human NK cells differentiate into CD56dim cells: role of contact with peripheral fibroblasts. J Immunol 179: 89–94. 17579025 10.4049/jimmunol.179.1.89

[14] De Maria A, Bozzano F, Cantoni C, Moretta L (2011) Revisiting human natural killer cell subset function revealed cytolytic CD56(dim)CD16+ NK cells as rapid producers of abundant IFN-gamma on activation. Proc Natl Acad Sci U S A 108: 728–732. 21187373 10.1073/pnas.1012356108 PMC3021076

[15] Fehniger TA, Cooper MA, Nuovo GJ, Cella M, Facchetti F, et al (2003) CD56bright natural killer cells are present in human lymph nodes and are activated by T cell-derived IL-2: a potential new link between adaptive and innate immunity. Blood 101: 3052–3057. 12480696 10.1182/blood-2002-09-2876

[16] Romagnani C, Juelke K, Falco M, Morandi B, D'Agostino A, et al (2007) CD56brightCD16- killer Ig-like receptor- NK cells display longer telomeres and acquire features of CD56dim NK cells upon activation. J Immunol 178: 4947–4955. 17404276 10.4049/jimmunol.178.8.4947

[17] Vitale M, Della Chiesa M, Carlomagno S, Romagnani C, Thiel A, et al (2004) The small subset of CD56brightCD16- natural killer cells is selectively responsible for both cell proliferation and interferon-gamma production upon interaction with dendritic cells. Eur J Immunol 34: 1715–1722. 15162442 10.1002/eji.200425100

[18] Warren HS, Kinnear BF, Kastelein RL, Lanier LL (1996) Analysis of the costimulatory role of IL-2 and IL-15 in initiating proliferation of resting (CD56dim) human NK cells. J Immunol 156: 3254–3259. 8617947

[19] Bjorkstrom NK, Ljunggren HG, Sandberg JK (2010) CD56 negative NK cells: origin, function, and role in chronic viral disease. Trends Immunol 31: 401–406. 20829113 10.1016/j.it.2010.08.003

[20] Zimmer J, Bausinger H, Andres E, Donato L, Hanau D, et al (2007) Phenotypic studies of natural killer cell subsets in human transporter associated with antigen processing deficiency. PloS One 2: e1033. 17940597 10.1371/journal.pone.0001033 PMC2001180

[21] Villa-Forte A, de la Salle H, Fricker D, Hentges F, Zimmer J (2008) HLA class I deficiency syndrome mimicking Wegener's granulomatosis. Arthritis Rheum 58: 2579–2582. 18668571 10.1002/art.23675

[22] Bielekova B, Catalfamo M, Reichert-Scrivner S, Packer A, Cerna M, et al (2006) Regulatory CD56(bright) natural killer cells mediate immunomodulatory effects of IL-2Ralpha-targeted therapy (daclizumab) in multiple sclerosis. Proc Natl Acad Sci U S A 103: 5941–5946. 16585503 10.1073/pnas.0601335103 PMC1458677

[23] Li Z, Lim WK, Mahesh SP, Liu B, Nussenblatt RB (2005) In vivo blockade of human IL-2 receptor induces expansion of CD56(bright) regulatory NK cells in patients with active uveitis. J Immunol 174: 5187–5191. 15843513 10.4049/jimmunol.174.9.5187

[24] Barcelos W, Sathler-Avelar R, Martins-Filho OA, Carvalho BN, Guimaraes TM, et al (2008) Natural killer cell subpopulations in putative resistant individuals and patients with active Mycobacterium tuberculosis infection. Scand J Immunol 68: 92–102. 18484953 10.1111/j.1365-3083.2008.02116.x

[25] Gineau L, Cognet C, Kara N, Lach FP, Dunne J, et al (2012) Partial MCM4 deficiency in patients with growth retardation, adrenal insufficiency, and natural killer cell deficiency. J Clin Invest 122: 821–832. 22354167 10.1172/JCI61014 PMC3287233

[26] Hughes CR, Guasti L, Meimaridou E, Chuang CH, Schimenti JC, et al (2012) MCM4 mutation causes adrenal failure, short stature, and natural killer cell deficiency in humans. J Clin Invest 122: 814–820. 22354170 10.1172/JCI60224 PMC3287227

[27] Beziat V, Descours B, Parizot C, Debre P, Vieillard V (2010) NK cell terminal differentiation: correlated stepwise decrease of NKG2A and acquisition of KIRs. PloS One 5: e11966. 20700504 10.1371/journal.pone.0011966 PMC2917352

[28] Bjorkstrom NK, Riese P, Heuts F, Andersson S, Fauriat C, et al (2010) Expression patterns of NKG2A, KIR, and CD57 define a process of CD56dim NK-cell differentiation uncoupled from NK-cell education. Blood 116: 3853–3864. 20696944 10.1182/blood-2010-04-281675

[29] Lopez-Verges S, Milush JM, Pandey S, York VA, Arakawa-Hoyt J, et al (2010) CD57 defines a functionally distinct population of mature NK cells in the human CD56dimCD16+ NK-cell subset. Blood 116: 3865–3874. 20733159 10.1182/blood-2010-04-282301 PMC2981540

[30] Sun JC, Lopez-Verges S, Kim CC, De Risi JL, Lanier LL (2011) NK cells and immune “memory”. J Immunol 186: 1891–1897. 21289313 10.4049/jimmunol.1003035 PMC4410097

[31] Beziat V, Duffy D, Quoc SN, Le Garff-Tavernier M, Decocq J, et al (2011) CD56brightCD16+ NK cells: a functional intermediate stage of NK cell differentiation. J Immunol 186: 6753–6761. 21555534 10.4049/jimmunol.1100330

[32] Dulphy N, Haas P, Busson M, Belhadj S, Peffault de Latour R, et al (2008) An unusual CD56(bright) CD16(low) NK cell subset dominates the early posttransplant period following HLA-matched hematopoietic stem cell transplantation. J Immunol 181: 2227–2237. 18641363 10.4049/jimmunol.181.3.2227

[33] Allan DS, Rybalov B, Awong G, Zuniga-Pflucker JC, Kopcow HD, et al (2010) TGF-beta affects development and differentiation of human natural killer cell subsets. Eur J Immunol 40: 2289–2295. 20540115 10.1002/eji.200939910 PMC3066635

[34] Smalley DM, Ley K (2005) L-selectin: mechanisms and physiological significance of ectodomain cleavage. J Cell Mol Med 9: 255–266. 15963248 10.1111/j.1582-4934.2005.tb00354.x PMC6740228

[35] Moldovan I, Galon J, Maridonneau-Parini I, Roman Roman S, Mathiot C, et al (1999) Regulation of production of soluble Fc gamma receptors type III in normal and pathological conditions. Immunol Lett 68: 125–134. 10397167 10.1016/s0165-2478(99)00041-3

